# Patterns of infectious complications in acute myeloid leukemia and myelodysplastic syndromes patients treated with 10‐day decitabine regimen

**DOI:** 10.1002/cam4.1231

**Published:** 2017-10-23

**Authors:** Alaa M. Ali, Daniel Weisel, Feng Gao, Geoffrey L. Uy, Amanda F. Cashen, Meagan A. Jacoby, Lukas D. Wartman, Armin Ghobadi, Iskra Pusic, Rizwan Romee, Todd A. Fehniger, Keith E. Stockerl‐Goldstein, Ravi Vij, Stephen T. Oh, Camille N. Abboud, Mark A. Schroeder, Peter Westervelt, John F. DiPersio, John S. Welch

**Affiliations:** ^1^ Department of Internal Medicine Division of Oncology Washington University Saint Louis Missouri; ^2^ Division of Public Health Sciences Department of Surgery Washington University St. Louis Missouri

**Keywords:** AML, decitabine, neutropenic fever

## Abstract

Decitabine has been explored as a reduced‐intensity therapy for older or unfit patients with acute myeloid leukemia (AML). To better understand the risk of infections during decitabine treatment, we retrospectively examined the culture results from each infection‐related serious adverse event that occurred among 85 AML and myelodysplastic syndromes (MDS) patients treated in a prospective clinical study using 10‐day cycles of decitabine at Washington University School of Medicine. Culture results were available for 163 infection‐related complications that occurred in 70 patients: 90 (55.2%) events were culture‐negative, 32 (19.6%) were gram‐positive bacteria, 20 (12.3%) were gram‐negative bacteria, 12 (7.4%) were mixed, 6 (3.7%) were viral, 2 (1.2%) were fungal, and 1 (0.6%) was mycobacterial. Infection‐related mortality occurred in 3/24 (13%) of gram‐negative events, and 0/51 gram‐positive events. On average, nearly one third of patients experienced an infection‐related complication with each cycle, and the incidence did not decrease during later cycles. In summary, in patients receiving 10‐day decitabine, infectious complications are common and may occur during any cycle of therapy. Although febrile events are commonly culture‐negative, gram‐positive infections are the most frequent source of culture‐positive infections, but gram‐negative infections represent a significant risk of mortality in AML and MDS patients treated with decitabine.

## Introduction

Decitabine is a cytosine analog with S‐phase‐dependent pharmacokinetics that is incorporated into DNA, but cannot be methylated 1, 2, 3. Historically, it has yielded clinical responses in ~25% of patients with acute myeloid leukemia (AML) or myelodysplastic syndromes (MDS) across diverse clinical trials, although response rates may be higher using a 10‐day versus 5‐day approach 4, 5, 6. In general, decitabine is well tolerated and can be given in the outpatient setting.

Decitabine treatment is associated with infection complications and readmission for neutropenic fevers 5, 7, 8. Limited data exist concerning the types of infections that occur in AML and MDS patients treated with decitabine.

We recently completed a study of AML and MDS patients treated with decitabine administered in 10 daily doses per cycle 5. Similar to others 6, we observed modestly higher response rates using the 10‐day regimen (46%) versus published results from 5‐day regimens (17–32%) 8, 9, 10, 11, and the majority of Common Terminology Criteria for Adverse Events (CTCAE)‐defined significant adverse events (SAEs) we observed were associated with infectious complications and readmission for neutropenic fevers 5. We also observed that response correlated with the presence of mutations in *TP53* 5.

Antibiotic prophylaxis has been controversial during AML chemotherapy (for both decitabine and cytarabine‐based treatments), and the benefits of prophylaxis are unclear.

In this study, we reviewed each infection‐related SAE observed among 85 patients to determine whether the infectious complications observed during decitabine treatment might be different from the complications of more intensive regimens, and whether specific bacteria were commonly observed that might be amenable to alternative prophylaxis.

## Patients and Methods

### Study design

We reviewed all CTCAE (version 4.0) grade 3–5 SAEs associated with infectious complications in 85 patients with AML or MDS who were enrolled between April 2013 and November 2015 on a clinical study at Washington University School of Medicine (NCT01687400) 5. Of these, 70 patients had infection‐related events with documented culture results that could be retrospectively evaluated. This study was approved by our institutional review board and conducted in accordance with the provisions of the Declaration of Helsinki. Response evaluation and molecular end‐points have been previously published 5.

### Treatment

Decitabine was administered intravenously at a dose of 20 mg/m^2^ of body surface area per day in 28‐day cycles. Initial cycles were given on consecutive days 1–10. Once patients achieved blasts <5%, they could reduce the dosing to days 1–5. After two cycles with dosing on days 1–5, the dose could be reduced further to days 1–3.

### Antimicrobial therapy

Prophylactic antimicrobial therapy was recommended, but not stipulated as part of the study. Recommended prophylaxis consisted of acyclovir, ciprofloxacin, and fluconazole. Patients who developed infection‐related complications were treated with antimicrobial and supportive therapy according to commonly accepted guidelines. The diagnostic work‐up in case of fever included collection of blood cultures from central venous catheters and peripheral vein and collection of nasal, pharyngeal, and anal swab. Standard chest radiographs were obtained at hospital admission and if clinically indicated. Cytomegalovirus (CMV) reactivation was not routinely tested. Prompt empiric first‐line therapies consisted of cephalosporin, penicillin, or carbapenem antibiotics. Vancomycin was added if fever persisted for 48–72 h and at least one of the following clinical findings was met: clinically suspected serious catheter‐related infections, known colonization with penicillin‐ and cephalosporin‐resistant pneumococci or methicillin‐resistant Staphylococcus aureus, positive blood cultures for gram‐positive bacteria before the final identification, hypotension or other evidence of cardiovascular impairment. An azole or echinocandin was given empirically if the patient did not respond to antibiotic therapy within 5–7 days. Modifications of the antibiotic therapy were made on the basis of results of cultures and susceptibilities of microorganisms to antimicrobial agents.

### Assessment of infection incidence and outcome

Each recorded infection‐related serious adverse event was reviewed. Fever was considered as a single temperature measurement of 38.4°C or 38.0°C over at least 1 h, in the absence of obvious environmental causes.

Neutropenia was defined as an absolute neutrophil count (ANC) less than 1.0 × 10^9^/L. ANC was further categorized according to whether they were <0.5 × 10^9^/L or <0.1 × 10^9^/L at the onset of febrile episodes.

The causative agent, when identified, was classified as bacterial (gram‐positive, gram‐negative, or mycobacterial), fungal (*Candida* species, *Aspergillus* species, or other), or viral (varicella zoster virus, herpes simplex virus [HSV]).

The site of infection was classified as blood (bacteremia), skin and/or soft tissues, catheter‐related blood stream infection, gastrointestinal (GI) tract, upper respiratory tract (pharyngitis, sinusitis, rhinitis), lower respiratory tract (pneumonia or bronchitis), urinary tract or joint/bone (septic arthritis, osteomyelitis).

The outcome of infections for each patient was determined at the completion of the antimicrobial therapy. All patients who died of infection were considered a failure.

### Statistical analysis

Data were analyzed through November 2016. Statistical analysis was performed using SPSS program, version 24 (IBM, Chicago, IL). Univariate analysis (Fisher exact test and Pearson chi‐square T‐test) was used for assessment of infection incidence. *P *<* *0.05 was considered statistically significant. The distribution of overall survival was described using Kaplan–Meier curves and compared by log‐rank test. Survival analysis was performed using Prism 5 (GraphPad, San Diego, CA).

## Results

### Patients

A total number of 282 cycles of decitabine were administered to 85 patients with AML or MDS (Table 1). Fifty patients were males (58.8%) and 35 were females (41.2%). The median age of the patient population was 74 years (range: 31–89 years). The median number of cycles received was 2 (range 0.5–17). Two hundred and forty‐eight hospital admissions following the administration of decitabine were reviewed. Of these admissions, 65.7% were for febrile episodes or infections (total of 163 admissions). The 30‐day mortality was 2.4%, the 60‐day mortality was 19%, and the 6‐month survival was 63.5%. The clinical features of patients with and without infection‐related events are shown in Table 1.

**Table 1:** Clinical data

Characteristic	Number of infection‐related SAEs				Totals
	0	1	2	>2	
Gender					
Male	12	18	8	12	50
Female	2	16	8	9	35
Age					
<40	0	1	0	1	2
40–60	0	4	2	1	7
>60	14	29	14	19	76
Performance status					
0	5	11	6	4	26
1	5	13	9	13	40
2	4	9	1	4	18
Unknown	0	1	0	0	1
Disease					
AML	13	19	11	15	58
MDS	1	15	5	6	27
Response					
CR	2	4	1	2	9
CRi/mCR	5	13	8	10	36
PR/SD/PD	3	11	5	8	27
Not evaluable	4	6	2	1	13
Cycles completed					
<1	4	4	2	1	11
1	2	8	3	2	15
2	4	8	6	4	22
3	0	4	3	4	11
>3	4	10	2	10	26
Total	14	34	16	21	85

AML, acute myeloid leukemia; MDS, myelodysplastic syndromes; SAEs, significant adverse events.

### Effect of clinical factors

Clinical features were correlated with the incidence of infection‐related SAEs (gender, age, performance status, disease, response, and cycles completed). The median and the mode number of infection‐related SAEs per patient in each subgroup was 1 (Table 1).

**Figure 1:**
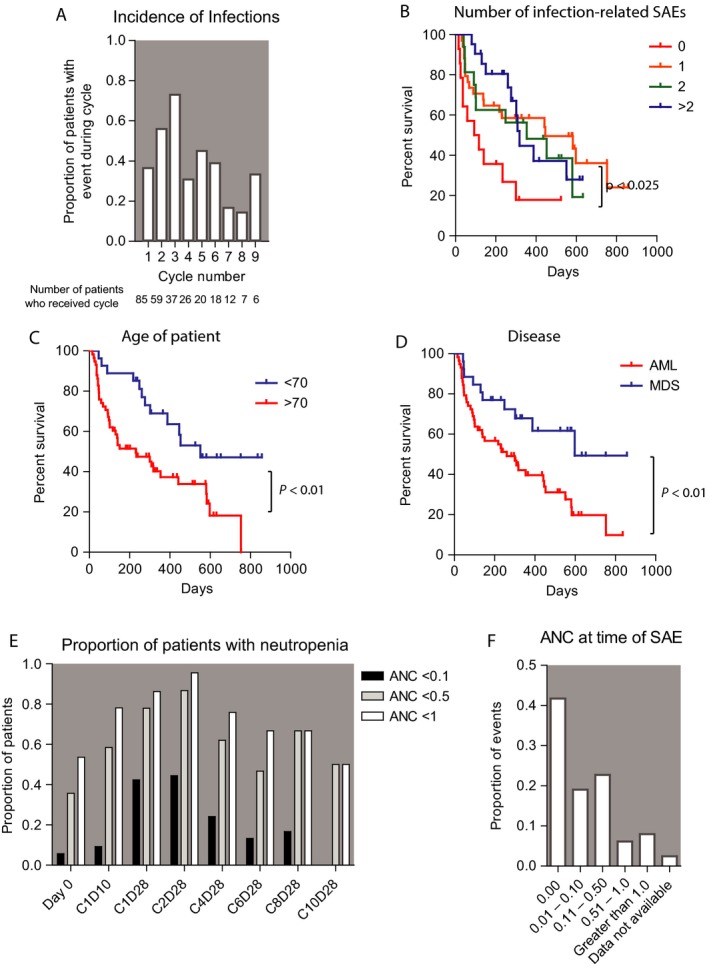
Correlation of infection events with clinical features. (A) The incidence of infection‐related events occurring within each cycle of therapy, restricted to cycle numbers received by at least five patients. (B) Overall survival correlation with the number of infection‐related SAEs a patient experienced during therapy. (C and D) Correlation of age and disease with overall survival. *P* values indicate results of log‐rank test. (E and F) Absolute neutrophil count (ANC) at the schedule bone marrow biopsies and at the time of infection‐related SAEs (ANC values indicate 10^9^ neutrophils per liter).

The incidence of infection‐related SAEs was correlated with the cycle number (Fig. 1A). On average, within each cycle, approximately one third of patients receiving that cycle experienced an infection‐related SAE, and we observed no consistent reduction in incidence during higher numbered cycles (i.e., cycles 4–9). Overall, the median survival of the cohort was 318 days. Counterintuitively, patients without an infection‐related SAE had shorter survival (median 104 days vs. 446, 354, and 318 days for patients with 0, 1, 2, or 3 or more SAEs, respectively), perhaps because they did not remain on therapy long enough to experience a complication (Fig. 1B).

In a univariate analysis comparing patients who had no infections versus those who had one or more, patients with younger age (age <70) tended to have a higher incidence of infections. However, older patients had shorter survival, and this may account for the disproportionate number of older patients without an infection‐related event (Fig. 1C). Nevertheless, the difference in infection incidence between the two groups (>70 vs. <70) did not reach a statistical significance (*P *=* *0.129). Likewise, compared to infections incidence in AML patients (77.5%), a higher incidence of infections was noted in MDS patients (96.3%, *P *=* *0.032). However, AML patients also had shorter survival compared with MDS patients (Fig. 1D). Therefore, patients who lived longer and had more therapy tended to have more infection episodes, although developing multiple infections did not affect the overall survival compared to patients who only experienced one or two infections (Fig. 1B).

Blood count results were prospectively recorded on day 0, cycle 1 day 10 (C1D10), cycle 1 day 28 (C1D28), and at the end of even cycles. Neutropenia was common across all time‐points (Fig. 1E). In total, 23%, 60%, and 69% of patients had an ANC <0.1, <0.5, and <1.0 at two or more measurements, respectively. At the time of each infection‐related SAE, the ANC was retrospectively assessed. The majority of patients had neutropenia with ANC <1.0 × 10^9^/L (88.6%), or ANC <0.5 × 10^9^/L (83%) at the onset of infection, and more than half of the patients (60.3%) presented with ANC <0.1 × 10^9^/L (Fig. 1F).

For each event, the recorded admission for antibacterial, antiviral, and antifungal prophylactic agents were reviewed (Table 2). In the majority of admissions (74.8%, 122 of 163), the patients were documented as receiving antiviral prophylaxis. Fewer patients were documented as receiving antibacterial (54%) or antifungal prophylaxis (41.7%). Of the admissions where patients were already receiving antibacterial prophylaxis, only 30% (27 of 90) had positive cultures for bacteria. In contrast, positive cultures were noted in 42.8% (33 of 77) of the admissions of patients with no antibacterial prophylaxis (*P* value: 0.08). Furthermore, two of the six positive cultures for fungi occurred in the setting of antifungal prophylaxis and five of the eight viral infections occurred in patients receiving antiviral prophylaxis (parainfluenza, HSV and rhinovirus/enterovirus).

**Table 2:** Prophylaxis at the time of infection

Prophylaxis	Number events	Class of prophylaxis	Totals
Ciprofloxacin	74	Total antibiotics	88 (54%)
Other antibiotics	14		
Acyclovir	101	Total antivirals	122 (74.8%)
Other antivirals	21		
Fluconazole	51	Total antifungals	68 (41.7%)
Other antifungal	17		

### Causative agent

In 55.2% of the admissions (90 of 163), a clinically suspected infection was associated with negative culture results. A causative agent of infection was documented in 73 (44.8%) of 163 admissions: gram‐positive infections in 32 (19.6% of total admissions), 20 (12.3%) were gram‐negative, 12 (7.4%) of admissions were associated with mixed infections (two or more different pathogens), 6 (3.7%) were viral, two (1.2%) were fungal, and 1 (0.6%) was mycobacterial. A total number of 91 pathogens were retrieved from the infectious diagnostic workups (cultures, swabs, etc. that were performed in these 73 admissions) (Table 3). Gram‐positive bacteria represented 56% of the total isolated pathogens: coagulase‐negative staphylococci including *Staphylococcus epidermidis* represented the most common gram‐positive organisms (23/91: 25.3%) followed by vancomycin‐resistant enterococcus (VRE) (10/91: 11%). *Pseudomonas* species (8/91: 8.8%) followed by *Escherichia coli* (6/91: 6.6%) were the most frequent gram‐negative pathogens. In the study population, 6 (6.6%) and 8 (8.8%) of the microbiology‐documented infections were caused by fungal and viral agents respectively.

**Table 3:** Causative agent associated with infection

Infection	Events	Totals (%)
Gram‐positive		51/91 (56)
MRSA	2	
Coagulase‐negative staph		
*Staphylococcus epidermidis*	8	
Others	15	
Enterococcus		
Vancomycin‐sensitive	2	
Vancomycin‐resistant	10	
Other		
Viridans group	4	
Corynebacterium	5	
Lactobacillus	2	
*Clostridium difficile*	3	
Gram‐negative		24/91 (26.4)
Pseudomonas	8	
*Escherichia coli*	6	
Enterobacteriaceae	2	
Stenotrophomonas	4	
B. fragilis	1	
Achromobacter	1	
Citrobacter	2	
Mycobacteria		2/91 (2.2)
M. gordonae	1	
*Mycobacterium abscessus*	1	
Fungus		6/91 (6.6)
Candida	2	
Aspergillus	1	
Mucor	1	
Fusarium	1	
Curvularia	1	
Viruses		8/91 (9)
Rhinovirus/enterovirus	3	
Parainfluenza virus	2	
HSV	1	
Adenovirus	1	
Coronavirus	1	

MRSA, methicillin resistant; HSV, herpes simplex virus.

### Site of infection

Lower respiratory tract infections were the most common site for infection (28 events: 17.2% of all admissions). This was followed by bacteremia (27 events: 16.6% of all admissions) (Table 4).

**Table 4:** Site of infection

Site	Events	Percent of admissions
Lower respiratory	28	17%
Peripheral blood	26	16%
Central line	27	16.5%
Wound or soft tissues	14	9%
GI	7	4.2%
Upper respiratory	6	4%
Urinary	3	1.8%

GI, gastrointestinal.

### Type of catheter

To understand whether indwelling catheters might influence the frequency and types of infections, we reviewed admission chest X‐rays and procedure notes associated with all 163 hospitalizations. In 101 of the 163 hospitalizations (62%), the patients had a central venous catheter (Hohn). Twenty (12%) had implanted port, 20 (12%) had peripherally inserted central catheters (PICC), and 22 (14%) had peripheral access. Patients who had Hohn or PICC catheters tended to have more gram‐positive infections compared to patients with other types of access (port and peripheral access). However, this correlation did not reach statistical significance (*P *=* *0.47).

### Infection susceptibility

Of the gram‐positive organisms identified, vancomycin and linezolid were most frequently associated with susceptibility. In contrast, gram‐negative organisms were most frequently susceptible to cefepime, meropenem, and gentamycin. Cefepime and vancomycin were the most commonly used empiric antibiotics overall (56.25% of the admissions). Ten of the *Enterococcus* cases (out of 12: 83.3%) were VRE. Two of the *Staphylococcus* cases (out of 25: 8%) were methicillin resistant (MRSA). One of the *E. coli* infections was caused by extended‐spectrum beta‐lactamase (ESBL)‐producing organism. A prophylactic antibacterial (ciprofloxacin 500 BID) was being administrated in five cases (38.5%) of the 13 cases of resistant microorganisms (MRSA, VRE, and ESBL) prior to admission. All the *Pseudomonas* infections were sensitive to carbapenems.

### Infection outcome

Complete recovery from infectious complications was observed in the majority of patients. The patients were discharged on a prophylactic antibiotic (ciprofloxacin 500 mg BID) in 37.5% of the admissions. The length of prophylaxis was indefinite in almost half of these cases.

During the study period, 10 (31.2%) of the gram‐positive and 3 (15%) of the gram‐negative infections required readmission within 6 weeks. A total of seven deaths were attributed to infection‐related complications, with an overall incidence of 8.2% (7 of 85 patients). Three deaths were due to gram‐negative bacteremia/sepsis (caused by *Pseudomonas* and ESBL‐producing *E. coli*), two due to pneumonia and acute respiratory failure, one due to mixed bacteremia (*Mycobacterium abscessus* and VRE), and one due to pneumonia associated with disseminated *fusarium*. Only one of these seven cases was on prophylactic antibiotics prior to admission.

### Effect of quinolone prophylaxis

Patients who were receiving quinolone prophylaxis at the time of the SAE tended to have fewer positive cultures compared to those who were not receiving prophylaxis (43% vs. 57%); although this did not reach a statistical significance (*P *=* *0.084). The presence of antibiotics prophylaxis at the time of admission was not associated with statistically significant higher rates of resistant microorganisms (*P *=* *0.087) or lower rates of mortality (*P *=* *0.705). Two of the cases of *Clostridium difficile* colitis occurred in the setting of prophylactic ciprofloxacin treatment, and one did not.

## Discussion

Relatively few precise data are available on the incidence and characteristics of bacterial, fungal, and viral causes of infections in AML and MDS patients treated with decitabine 12, 13. In this study, we investigated retrospectively the infectious complications that occurred in 85 patients with AML or MDS enrolled in NCT01687400. In this study, we have described the prevalence and characteristics of clinically and microbiologically defined infections and infection‐related mortality, and the relationship between number of cycles and infection‐related events as well as the effect of prophylaxis on the positivity of cultures and susceptibility of microorganisms.

Neutropenia was common across all cycles of therapy (Fig. 1E). Based on the frequency and duration of neutropenia observed in this cohort, current guidelines would recommend consistent quinolone prophylaxis and consideration of antifungal prophylaxis 14, 15.

Infection‐related grade 3–5 SAEs occurred in 70 of 85 patients (82%) during therapy, and were observed in approximately one third of patients during each cycle. When compared to the incidence of infections when using a 5‐day decitabine regimen (29%) 9, longer courses of therapy (10‐day) were associated with more infections: 82% in this study and 68% reported by Blum et al. 6. However, 30‐day mortality and 6‐month survival remained comparable (2.4% vs. 7% and 63.5% vs. 60% in this cohort and a cohort treated with the 5‐day regimen, respectively) 9.

Fever and infections were the most frequent cause of hospitalization in our patient population. The majority of these events were not associated with a causative agent; an identifiable organism was isolated in 45% of admissions and bacteria represented the most commonly identified source of infection. Microbiologically or clinically documented fungal or viral infections were diagnosed in less than 5% of hospitalizations. In our study, the ratio of gram‐positive to gram‐negative bacteria approached 2:1. Gram‐positive cocci (predominantly coagulase‐negative staphylococci) represented the most frequently isolated microorganisms. This finding is not surprising, and consistent with the literature of other chemotherapeutic agents in hematological malignancies as well as solid cancers 16, 17. This dominant gram‐positive pattern can be attributed to the widespread use of indwelling intravascular catheters and the use of prophylaxis targeted against gram‐negative organisms (e.g., ciprofloxacin). Surgically implanted tunneled catheters have been associated with lower rates of bloodstream infections than percutaneously inserted catheters 18, 19. In this study, we observed a nonsignificant trend toward greater numbers of gram‐positive infections in patients with Hohn and PICC catheters, as might be expected. AML and MDS patients receiving decitabine frequently present with cytopenias and are unable to obtain a surgically implanted catheter until they achieve remission. This limits this opportunity for alternative access, and physicians should be cognizant of the risks and opportunities posed by different forms of percutaneous access. Enterococcal species, especially vancomycin‐resistant bacteria, represented the second most common gram‐positive isolates in our population, causing mainly bacteremia and urinary tract infections. Nevertheless, the rate of VRE infections appears to be similar in our patients compared to patients undergoing induction with more intensive chemotherapy (11% compared to 10%) 20, 21. Although colonization is far more frequent than true infection even in immunocompromised patients, bacteremia with VRE has been involved in outbreaks among oncology patients treated with chemotherapy and can be associated with increased mortality rates 20, 22.

Viridans group streptococci are other important pathogens that are classically seen in patients undergoing induction with high‐dose intensive chemotherapy associated with neutropenia and oropharyngeal mucositis 23, 24, 25, 26. Four cases of this type of bacteremia have been isolated in our patient group (2.5%). Only one case had mucositis (rare with decitabine) at the time of bacteremia. Therefore, other ports of entry, such as GI mucosa or central venous lines, may represent potential sources for these infections in these patients.

Infection‐related mortality in our cohort was associated with gram‐negative bacteremia, pneumonia, and one case of disseminated *Fusarium*. These data are consistent with historical outcomes of aerobic gram‐negative bacilli (*E*. *coli* and *Pseudomonas aeruginosa*) 27, 28, and the focus of our antibacterial prophylaxis targeted these bacteria. Primary prophylaxis with fluoroquinolones in our patient population was associated with a trend toward fewer positive blood cultures (*P *=* *0.084), but was not associated with higher rates of resistant microorganisms or lower rates of mortality.

Fungal infections are an infrequent, but recurrent cause of morbidity and mortality. We observed an incidence of probable/proven invasive fungal infections of 7% including the mixed infections. Although the spectrum of the fungal pathogens is similar (commonly, *Aspergillus* species and *Candida* species), the incidence may be modestly lower than the reported 12% incidence rate of IFIs in AML patient treated with more intensive cytotoxic therapy 29. Nevertheless, considering the high number of observed clinically suspected infections with negative cultures, we cannot exclude that the incidence of invasive fungal infections might be higher.

Similar patterns of infectious complications have been reported in AML patients during intensive chemotherapy. A review of 747 adults with AML treated with conventional chemotherapy identified a source organism in 56.6% of the febrile episodes 30. Although bacterial infections were also the most frequent cause of infectious complications (38.4%), more gram‐negative infections were observed (ratio 1:1), and a modestly higher incidence of fungal infections was observed (13.8%) 29, 30.

In sum, we found that infection‐related SAEs were common in AML and MDS patients undergoing a 10‐day decitabine regimen. In this cohort, admission to the hospital for evaluation of neutropenic fever occurred in the majority of patients at some point during treatment, and occurred in nearly one third of patients during each cycle of treatment. A causative agent was identified in less than half of admissions, and gram‐positive bacteria were the most frequent agents identified. However, gram‐negative infections were associated with mortality. Although expanding antimicrobial prophylaxis to include better gram‐positive coverage (e.g., ampicillin or augmentin) could reduce the incidence of hospital admissions, this is unlikely to have an impact on overall survival. Furthermore, the downsides of this approach including toxicities and the potential for promoting resistance should be considered. Prospective studies are needed to determine whether the benefit of antibacterial prophylaxis outweighs the risks.

## Conflict of Interest

The authors declare no conflicts of interest.
